# A young Botswana patient with congenital iris ectropion uvea

**DOI:** 10.11604/pamj.2016.25.42.10593

**Published:** 2016-09-29

**Authors:** Jemal Zeberga Shifa, Othokawa Nkomazana, Negussie Alula Bekele, Mamo Woldu Kassa

**Affiliations:** 1 Department of Surgery, Faculty of Medicine, University of Botswana, Gaborone, Botswana; 2 Department of Anaesthesia and Critical care Medicine, Faculty of Medicine, University of Botswana, Gaborone, Botswana

**Keywords:** Congenital, iris ectropion, glaucoma

## Abstract

Congenital iris ectropion is a rare condition; non-progressive anomaly characterised by the presence of iris pigment epithelium on the anterior surface of the iris stroma and is frequently associated with anterior iris insertion, dysgenesis of the drainage angle and glaucoma. This paper describes unusual case of bilateral case of congenital iris ectropion in adult patient with pupillary abnormality, normal anterior chamber angle structure and with no evidence of glaucoma.

## Introduction

Ectropion uvea is defined as the presence of iris pigment epithelium on the anterior surface of the iris. Although ectropion uvea typically is an acquired condition, it may also occur as an isolated congenital anomaly or in association with systemic disease such as neurofibromatosis [1]. Congenital ectropion uvea is a rare disorder, and epidemiological data on its incidence are scarce. Although bilateral cases have been reported, the disease is usually unilateral and non-progressive. Congenital iris ectropion is characterized by iris pigment hyperplasia onto the anterior surface of the iris around the pupillary margin. In contrast to acquired ectropion uvea, the iris sphincter muscle and stroma are not affected. The hyperplasia is thought to be induced by a primordial endothelium, an embryological remnant that fails to fully regress in the anterior chamber. This anomaly may be caused by a late developmental arrest of neural crest tissue in utero [1]. We present a case of bilateral congenital ectropion uvea without any visual complication in a young patient who has no sign of glaucoma.

## Patient and observation

**Figure 1:**
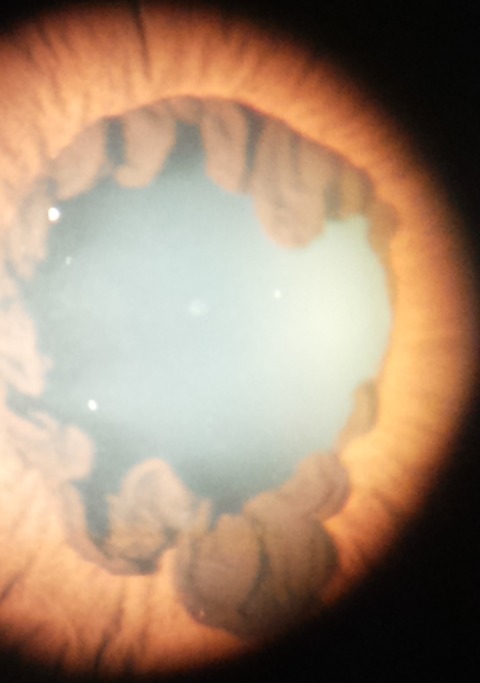
Hyperplasia of iris epithelium over anterior surface of the iris on left eye of the patient

A 24 years old boy presented to our eye clinic with a complaint of itching and tearing of both eyes of one month duration. This was his first time to visit the eye clinic. There was no eye discharge. There is neither family history of blindness nor associated systemic disease. He had occasional photophobia without any reduction of vision. On physical examination, the visual acuity in both eyes was 6/6 with cup to disc ratio of 0.3 on both sides. The intraocular pressure in the right was 14MM HG and left was 12 MMHG. The examination of iris showed glassy, smooth iris surface with proliferation of iris pigment epithelium on to anterior surface of the iris in both eyes (Figure 1). The pupillary examination showed round appearance, reactive to light but irregular in shape due to abnormal iris pigment epithelium. Visual Field Humphrey is normal. Goniscopy showed open angle and there is no anterior iris insertion in both eyes. Motility of the eyes is normal in all directions and there is no sign of ptosis in both eyes.

## Discussion

In the normal iris, the pigment epithelium forms a double layer that includes itself into the pupillary margins as the pigmented ruff. A congenital exaggeration of this process occurs where the pigmented layer advances over the anterior surface of the iris to a varying degree Glaucoma is the frequent complication of congenital ectropion uvea which was present in most cases reported. In one report, glaucoma was observed in seven of eight cases of congenital ectropion uvea. In the same study glaucoma was most commonly diagnosed in childhood or early adolescence, but the patient age at the time of diagnosis ranged from 7 months to 42 years. The variation in onset and severity of glaucoma may be explained by the degree of arrest in the iris migration and subsequent trabecular mesh work malformation [1–3]. In our case there is anterior growth of pigmented layer of the iris over anterior surface with sign of glaucoma is not yet observed but he needs close follow up by eye specialist for early signs of glaucoma. The extent to which the pupillary circumference involved in this case is variable [4, 5]. The pupil may be normal in size in some cases and larger than its counterpart in other. It may be round and reactive to light but may be oval and less reactive to light. The pupillary finding in our case is irregular in shape in both eyes involving the whole circumference and reactive to light. The iris stroma is generally hypoplastic to variable degree with smooth anterior surface with absence of central concentric furrows and radial folds. In our case there is loss of normal iris architecture. The condition is usually unilateral, but bilateral case has been reported like our case. The affected eye may exhibit mild to moderate ptosis with good levator function. This finding is most likely related to neural crest cell origin of Mueller’ muscle. The aetiology of this condition remains unknown but is probably due to imbalance of growth between the ectoderm and the mesoderm, usually a hyperplasia of the former. In contrast, acquired ectrpion uvea is often observed with neovascularization of the iris and neovascular glaucoma. It is also associated with any inflammatory, ischemic or neoplastic process involving the iris. This type of ectropion is commonly progressive. A contracting fibrovascular membrane with atrophy of the iris stroma causes a tractional curling of the posterior pigment epithelium, iris sphincter muscle, and stroma around the pupillary margin [4–6]. Some cases of congenital iris ectropion have been reported in the literature and most of them are associated with neurofibromatosis, Prader-Willi syndrome and Rieger anomaly [7]. In our case there is no association of systemic illness. Willcock and et al described 3 cases of bilateral ectropion uvea, and in 2 a link was found with the PAXE 6 gene Bilateral disease should be investigated for other possible causes [8].

## Conclusion

A Patient with iris ectropion may be overlooked so it so important that the need for high index of suspicion or referral linkage with tertiary hospitals with specialized Ophthalmological services. Although our case did not have Glaucoma at the time of diagnosis, it is likely that he might develop Glaucoma and other complications with time. As Glaucoma may develop at any stage, there has to be a regular screening for Glaucoma in congenital iris ectropion cases in order to recognise the complication early and provide timely Glaucoma management.
